# Supplementary material for: Cyclopropane-Containing Specialized Metabolites from the Marine Cyanobacterium cf. Lyngbya sp
Source: Molecules. 2023 May 8;28(9):3965. doi: 10.3390/molecules28093965 (PMC10180397; doi:10.3390/molecules28093965)
Supplement: Supplementary file 1 [file molecules-28-03965-s001.zip › molecules-2359379-supplementary.pdf]

Supplementary Information

Table of Content	Page
**Figure S1.** Incubation of VLC-derived fractions (fractions 1 to 8) at 100 μg/mL with *Pseudomonas aeruginosa* PAO1 *lasB-gfp* biosensor strain.	2
**Figure S2.** Mass spectra of VLC-derived fraction 3 (**A**) and fraction 4 (**B**) obtained by ESI-TOF-MS in positive ion mode, overlaid with the LC-UV chromatogram (light green lines).	2
**Table S1.** Mass spectral data and compound dereplication with the MarinLit database for compounds found in the LCMS analysis of fraction 3.	3
**Table S2.** SMILE notation and InChI code for **2**.	3
**Figure S3.** UV and HR-QTOF MS spectra of **2**.	4
**Figure S4.** ^{1}H NMR spectrum of **2** in $CDCl_3$.	4
**Figure S5.** ^{13}C NMR spectrum of **2** in $CDCl_3$.	5
**Figure S6.** DEPT spectra of **2** in $CDCl_3$.	6
**Figure S7.** HSQC NMR spectrum of **2** in $CDCl_3$.	7
**Figure S8.** COSY NMR spectrum of **2** in $CDCl_3$.	8
**Figure S9.** HMBC spectrum of **2** in $CDCl_3$.	9
**Figure S10.** NOESY spectrum of **2** in $CDCl_3$.	10
**Figure S11.** Molecular docking of (**A**) natural ligand, *N*-3-oxo-dodecanoyl-*L*-homoserine lactone, (**B**) pitinoic acid A (**8**), (**C**) dysidazirine carboxylic acid (**9**) and (**D**) majusculoic acid onto LasR-ligand binding domain performed on Swissdock.	11

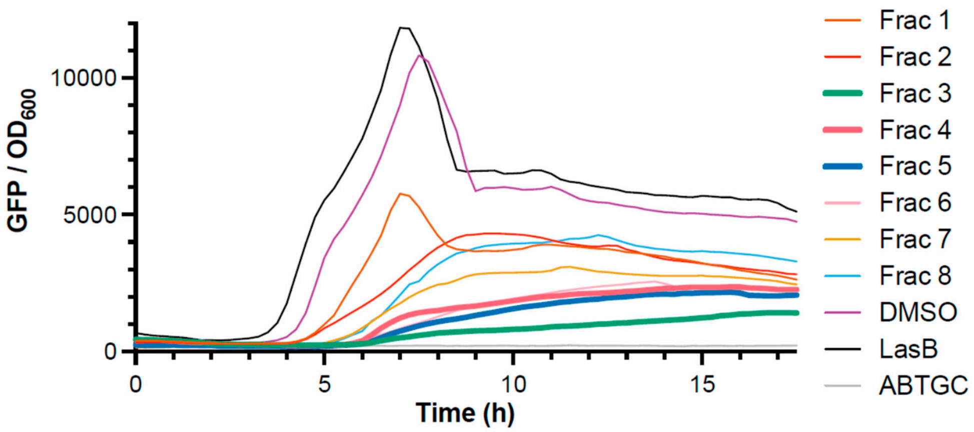

Figure S1. Incubation of VLC-derived fractions (fractions 1 to 8) at 100 µg/mL with *Pseudomonas aeruginosa* PAO1 *lasB-gfp* biosensor strain. GFP/OD_{600} values over a span of 17.5 h. DMSO, LasB, and ABTGC were used as negative, bacteria and media controls, respectively. The assay was conducted in triplicate at an effective concentration of 100 µg/mL. A lower GFP/OD_{600} indicates better QS inhibition. Fractions 3 to 5 demonstrated the best QS inhibition and are represented by the thicker lines.

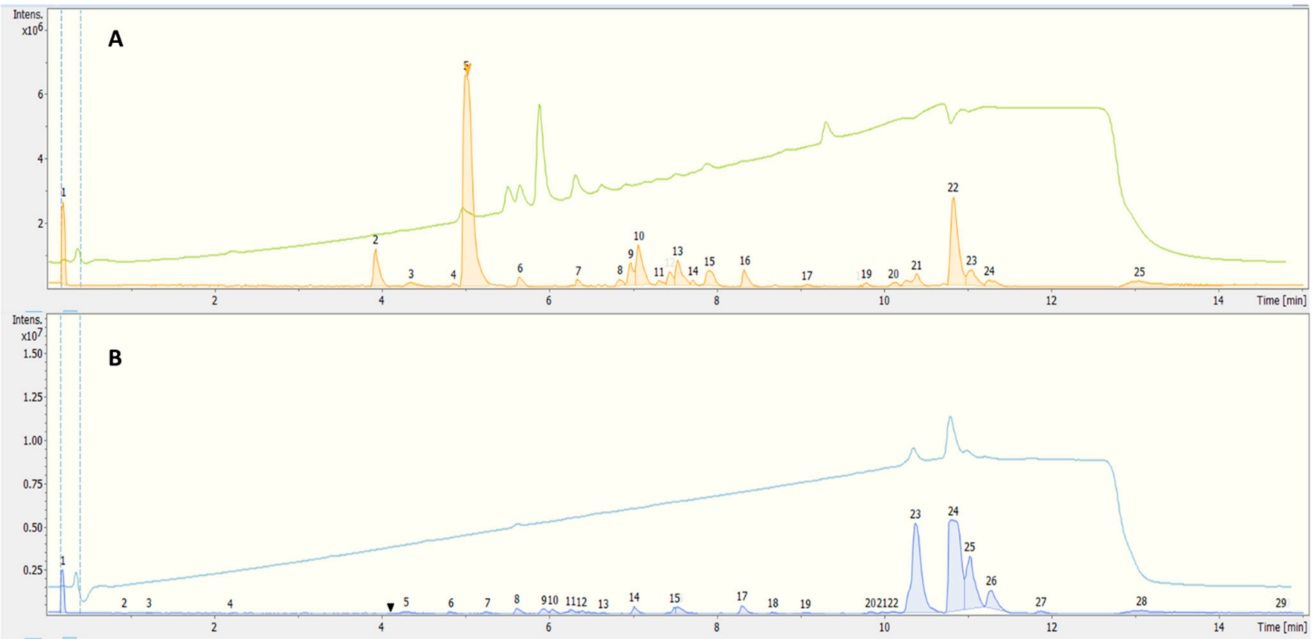

Figure S2. Mass spectra of VLC-derived fraction 3 (**A**) and fraction 4 (**B**) obtained by ESI-TOF-MS in positive ion mode, overlaid with the LC-UV chromatogram (light green lines).

Table S1. Mass spectral data and compound dereplication with the MarinLit database for metabolites found in the LCMS analysis of fractions 3 and 4.

Fraction	RT (min)	Measured Mass $[M+H]^+$	Predicted MF $[M+H]^+$	Err (in ppm)	Database search (Marinlit and Antibase)	Ref
3	5	279.0938	$C_{20}H_{11}N_2$	-4.4	Pukeleimide A or B	[1]
4	5.9	379.2435	$C_{17}H_{35}N_2O_7$	1.1	No hit	
3	6.4	261.1457	$C_{11}H_{21}N_2O_5$	-4.9	No hit	
4	7.0	271.2247	$C_{16}H_{31}O_3$	6.1	Malyngolide	[2]
3	7.1	347.2190	$C_{17}H_{27}N_6O_2$	-0.3	No hit	
3	7.4	349.2345	$C_{17}H_{29}N_6O_2$	0.4	No hit	
3	7.7	429.1603	$C_{21}H_{34}BrO_4$	0.8	No hit	
3	7.9	331.2240	$C_{16}H_{31}N_2O_5$	-1.6	Phomopsidin	[3]
3	9.8	257.2468	$C_{16}H_{33}O_2$	2.0	(-)-*trans*-7*S*-methoxytetradec-4-enoic acid	[4]
3, 4	9.8	609.2685	$C_{35}H_{37}N_4O_6$	3.7	No hit	
3, 4	9.8	637.3001	$C_{37}H_{41}N_4O_6$	2.3	No hit	
3	10.3	537.4872	$C_{32}H_{57}O_6$		No hit	
3, 4	10.4	623.2858	$C_{36}H_{39}N_4O_6$	0.7	Pheophorbide-like compound	[5]
3, 4	10.8	607.2916	$C_{36}H_{39}N_4O_5$	-0.1	No hit	
4	11.3	321.3128	$C_{22}H_{41}O$	5.0	No hit	

1. Cardellina, J.H.; Moore, R. E. The structures of pukeleimides A, B, D, E, F, and G. *Tetrahedron Lett.* **1997**, *20*, 2007–2010.
2. Cardellina, J.H.; Moore, R.E.; Arnold, E.V.; Clardy, J. Structure and absolute configuration of malyngolide, an antibiotic from the marine blue-green alga *Lyngbya majuscula* Gomont. *J. Org. Chem.* **1979**, *44*, 4039–4042.
3. Namikoshi, M.; Kobayashi, H.; Yoshimoto, T.; Hosoya, T. Phomopsidin, a new inhibitor of microtubule assembly produced by phomopsis sp.. isolated from coral reef in Pohnpei. *J. Antibiot.* **1997**, *50*, 890–892.
4. Cardellina, J.H.; Dalietos, D.; Marner, F.-J.; Mynderse, J.S.; Moore, R.E. (−)-trans-7(s)-methoxytetradec-4-enoic acid and related amides from the marine cyanophyte *Lyngbya majuscula*. *Phytochemistry* **1978**, *17*, 2091–2095.
5. Ohta, S.; Ono, F.; Shiomi, Y. et al. Anti-herpes simplex virus substances produced by the marine green alga, *Dunaliella primolecta*. *J. Appl. Phycol.* **1998**, *10*, 349–356.

Table S2. SMILE notation and InChI code for **2**.

**SMILE**	CCCCCCC[C@@H]1C[C@H]1CCC(OC/C=C/C(Br)=C)=O
**InChI**	1/C18H29BrO2/c1-3-4-5-6-7-10-16-14-17(16)11-12-18(20)21-13-8-9-15(2)19/h8-9,16-17H,2-7,10-14H2,1H3/b9-8+/t16-,17-/m1/s1/i1-12,2-12,3-12,4-12,5-12,6-12,7-12,8-12,9-12,10-12,11-12,12-12,13-12,14-12,15-12,16-12,17-12,18-12,19-79,20-16,21-16

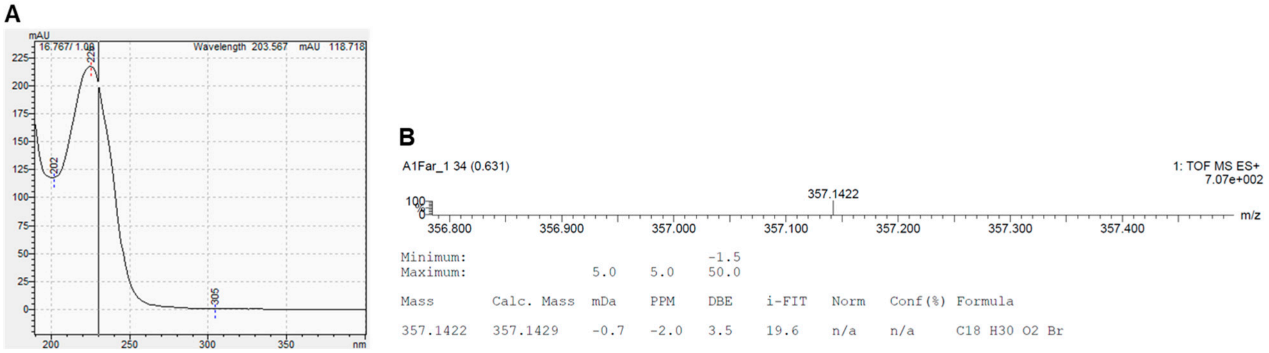

Figure S3. (**A**) UV and (**B**) HR-QTOF MS spectra of **2**.

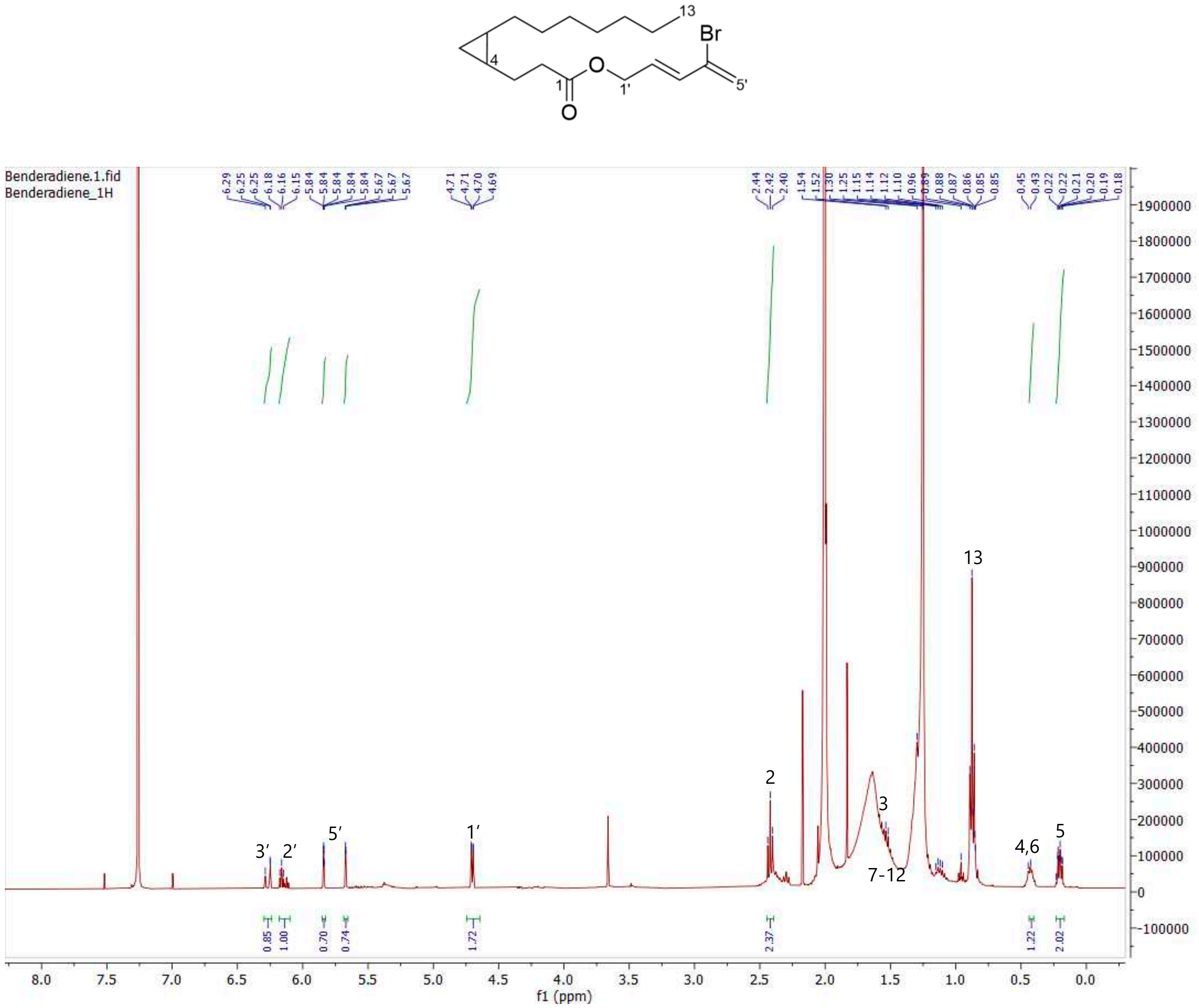

Figure S4. ^{1}H NMR spectrum of **2** in $CDCl_3$.

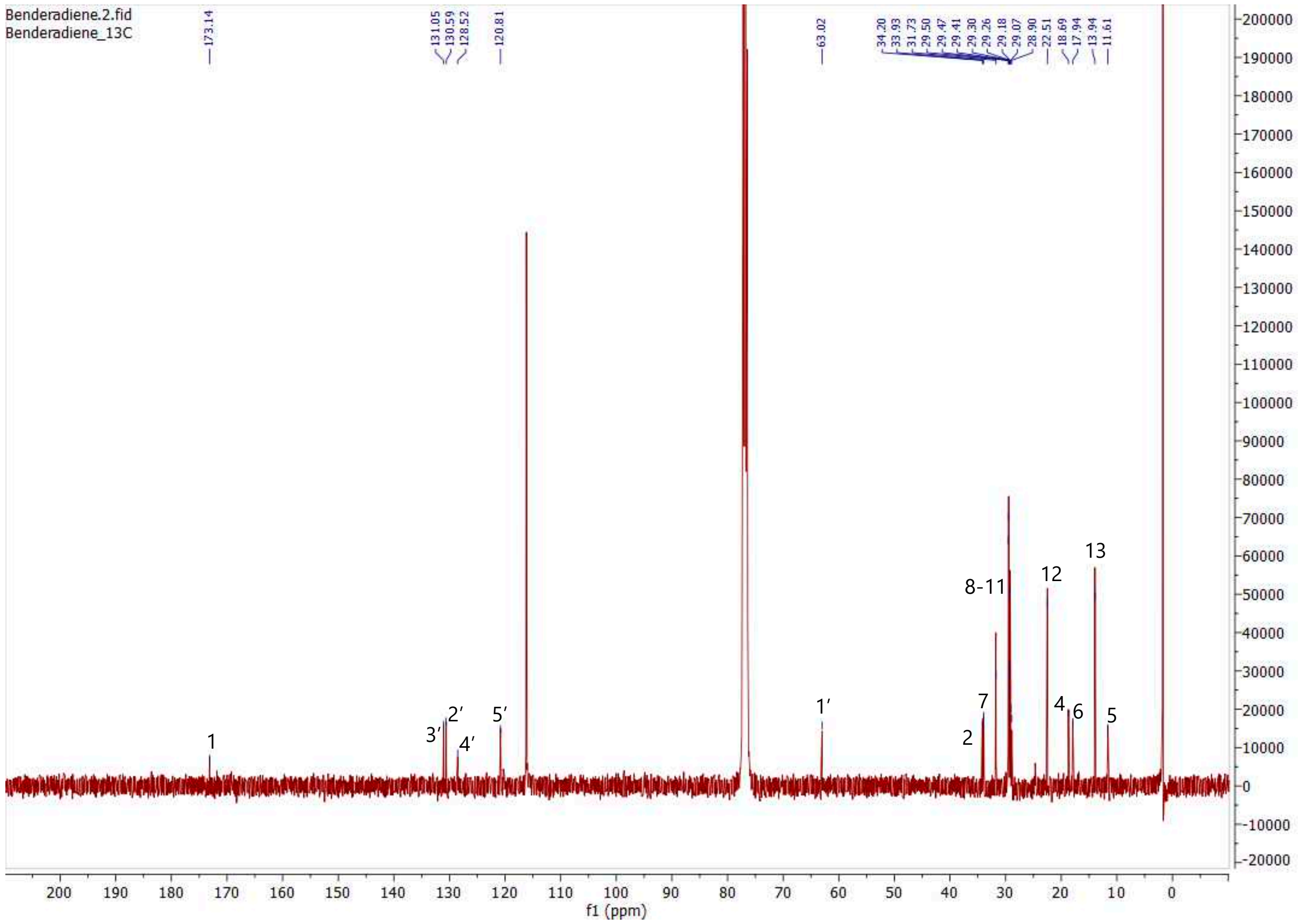

Figure S5. ^{13}C NMR spectrum of **2** in $CDCl_3$.

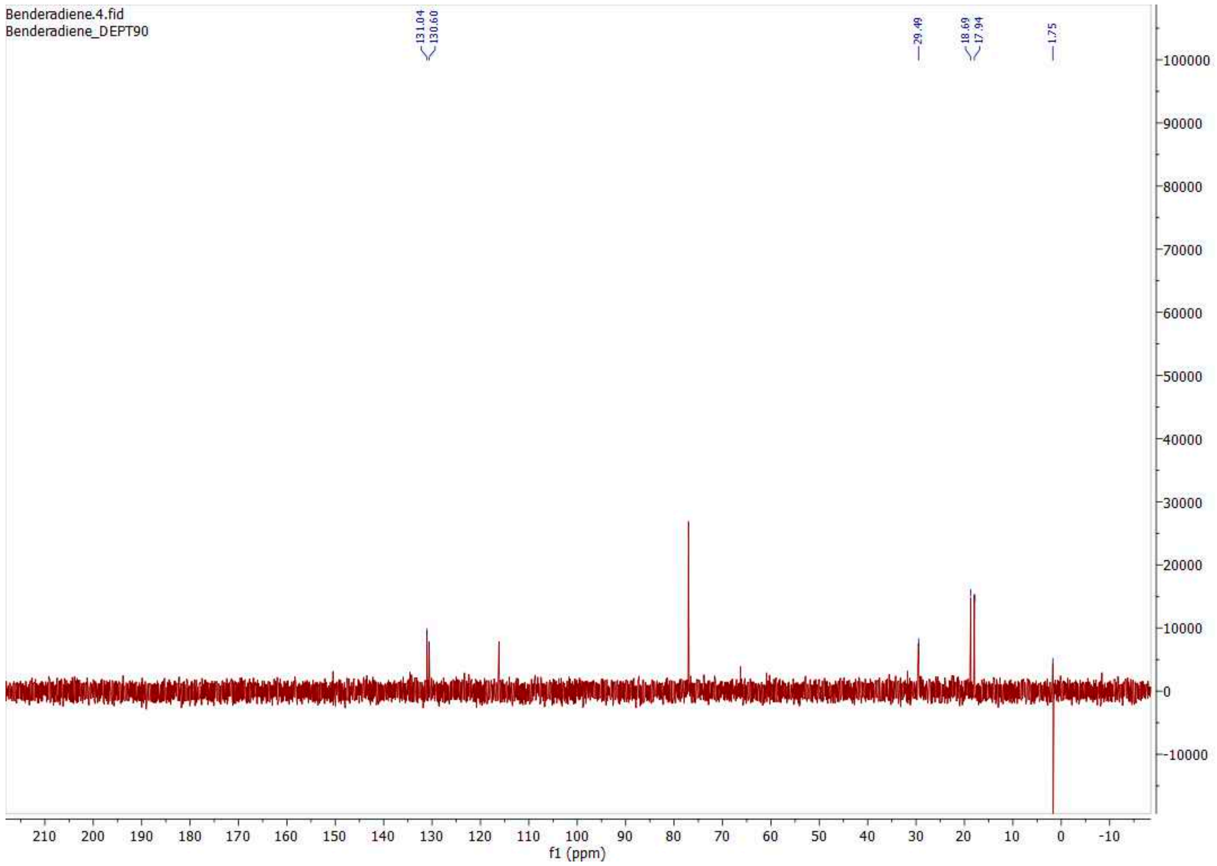

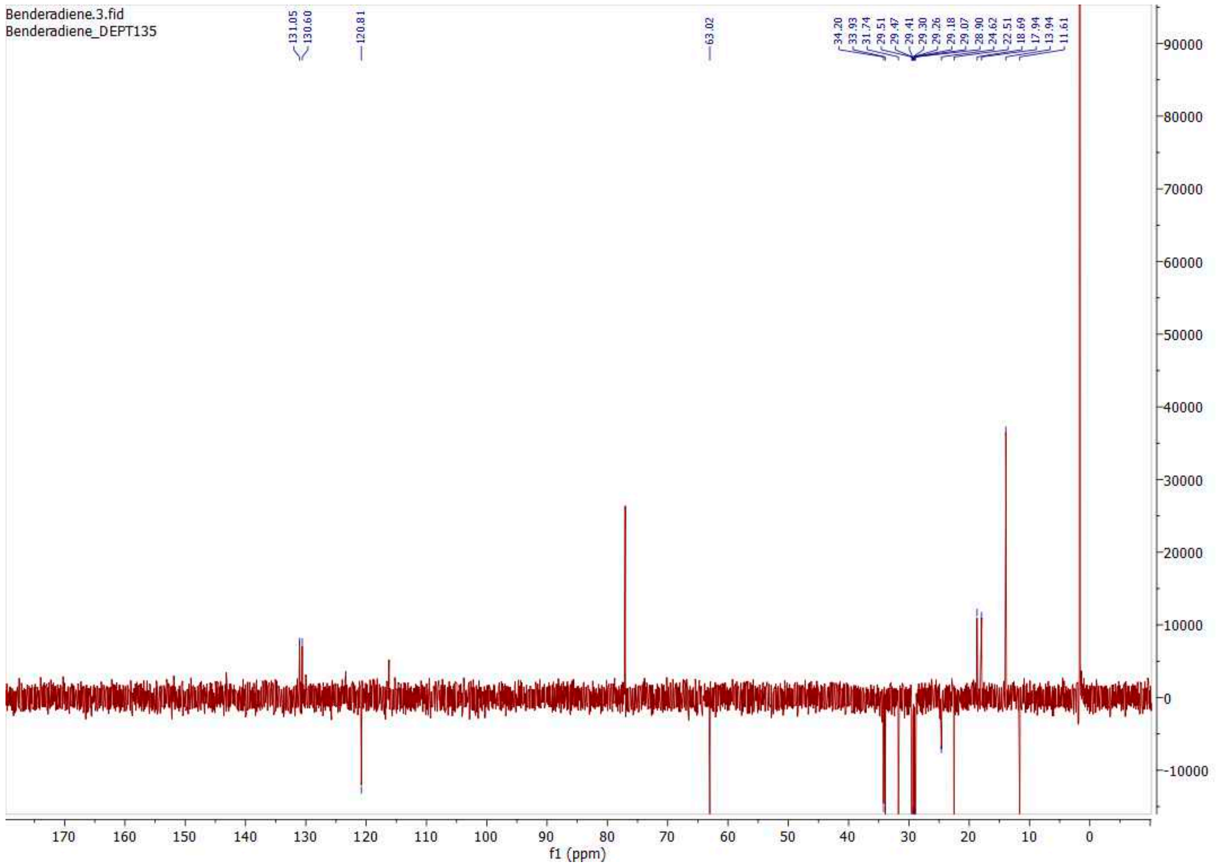

Figure S6. DEPT spectra of **2** in $CDCl_3$.

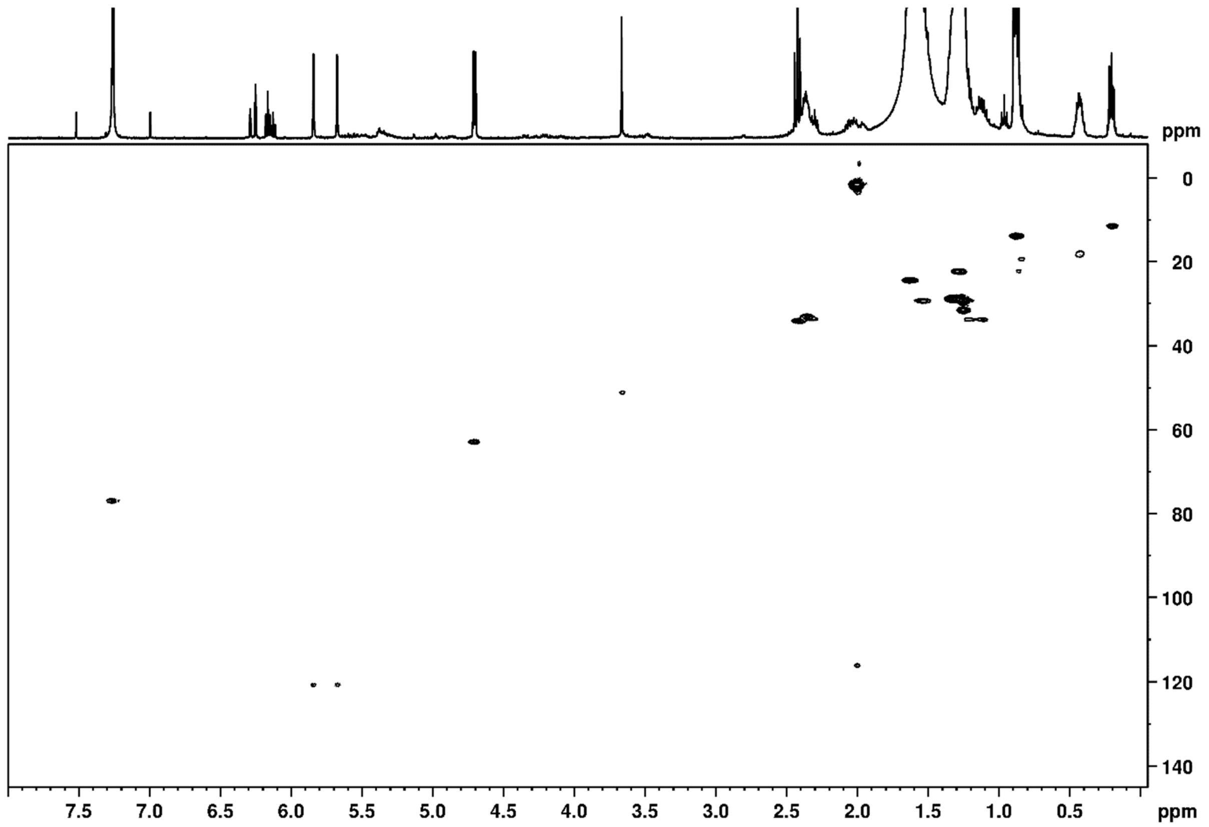

Figure S7. HSQC NMR spectrum of **2** in $CDCl_3$.

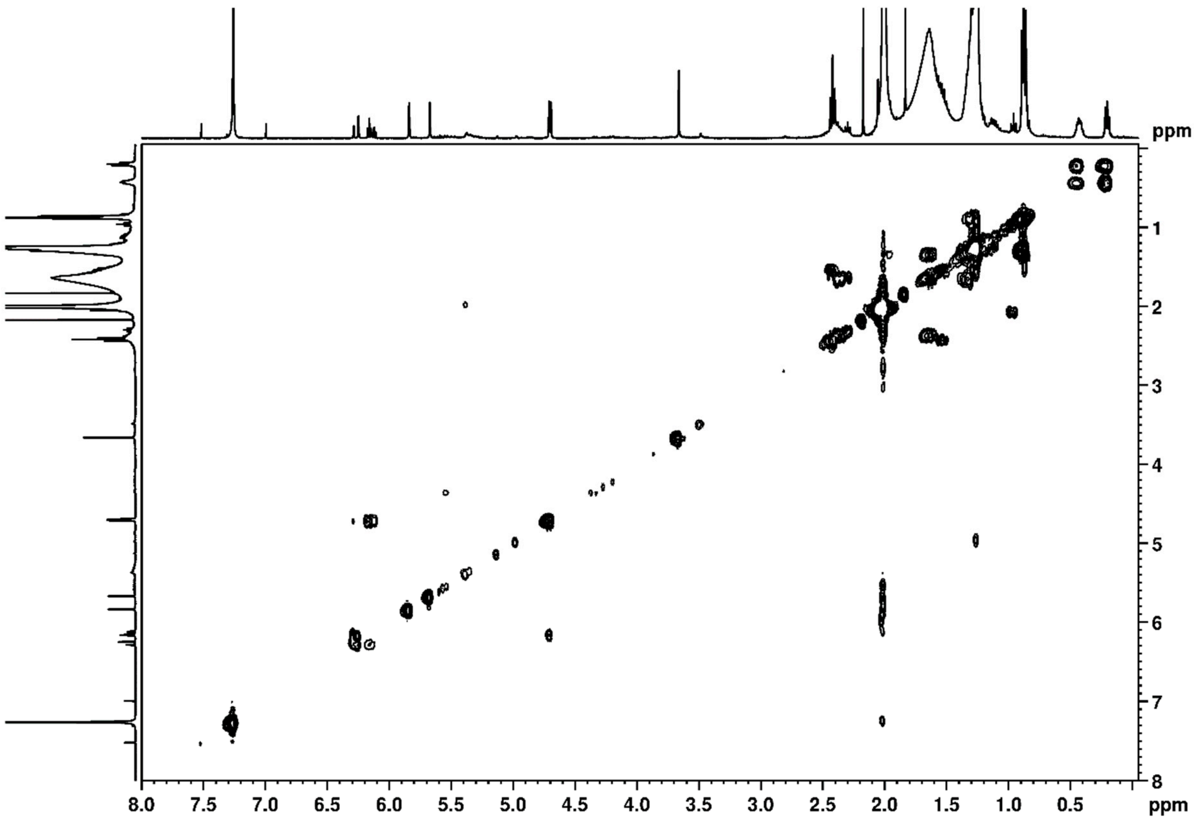

Figure S8. COSY NMR spectrum of **2** in $CDCl_3$.

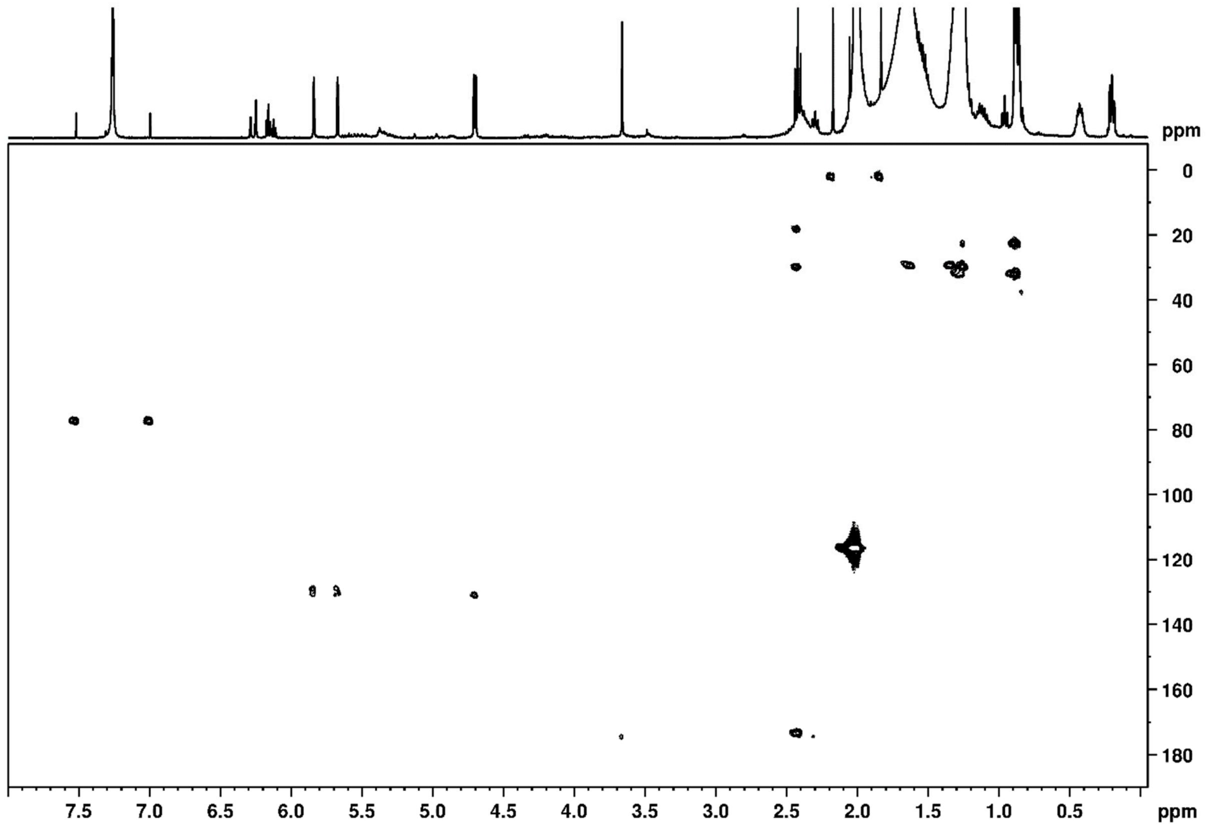

Figure S9. HMBC spectrum of **2** in $CDCl_3$.

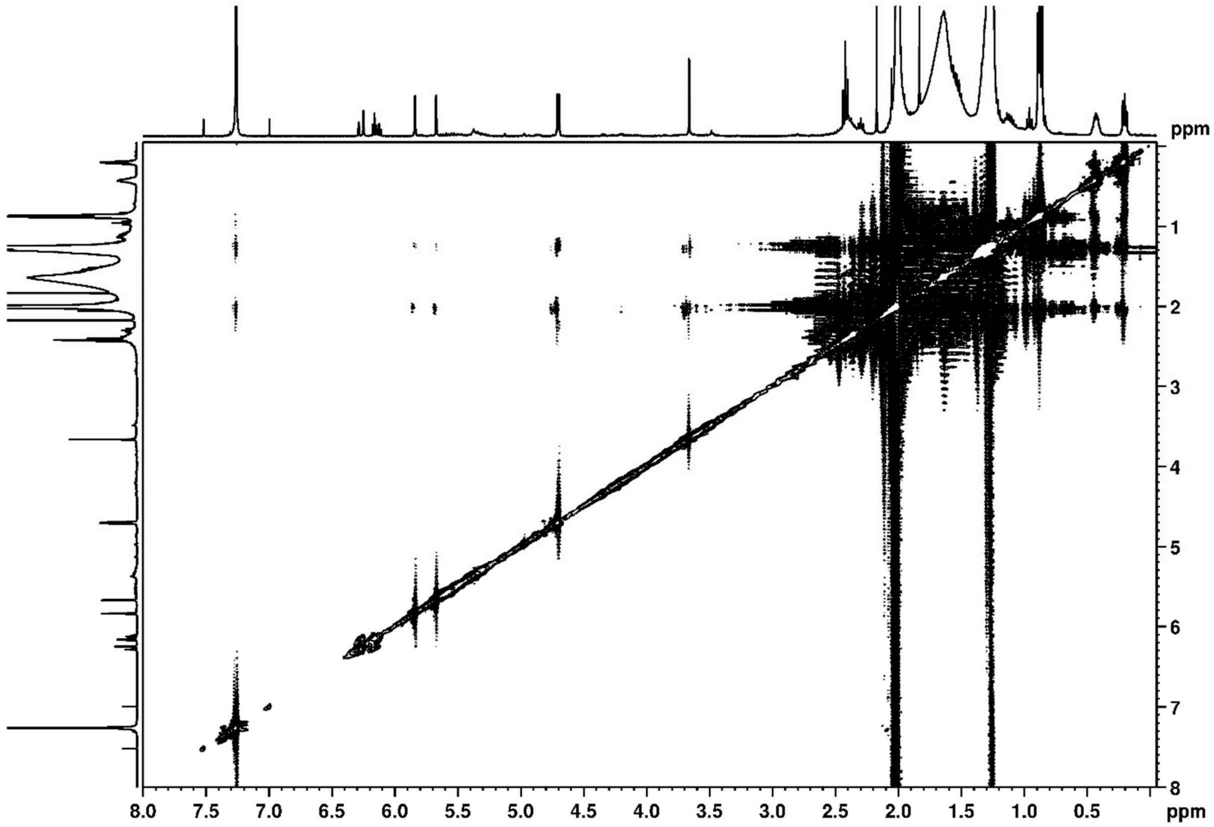

Figure S10. NOESY spectrum of **2** in $CDCl_3$.

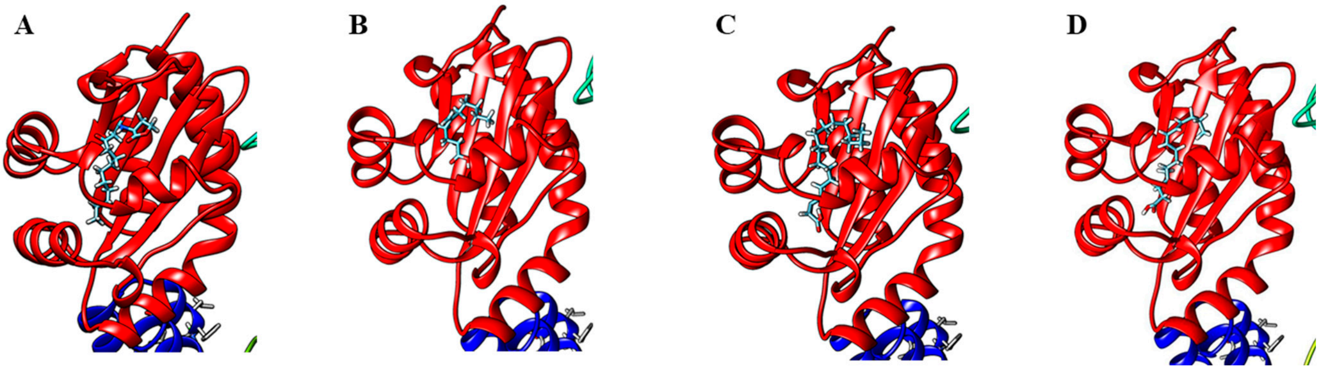

Figure S11. Molecular docking of (**A**) natural ligand, *N*-3-oxo-dodecanoyl-*L*-homoserine lactone, (**B**) pitinoic acid A (**8**), (**C**) dysidazirine carboxylic acid (**9**) and (**D**) majusculoic acid onto LasR-ligand binding domain performed on Swissdock.
